# Agomelatine-based *in situ* gels for brain targeting via the nasal route: statistical optimization, *in vitro*, and *in vivo* evaluation

**DOI:** 10.1080/10717544.2017.1357148

**Published:** 2017-07-26

**Authors:** Ahmed M. Fatouh, Ahmed H. Elshafeey, Ahmed Abdelbary

**Affiliations:** a Department of Pharmaceutics and Industrial Pharmacy, Faculty of Pharmacy, Cairo University, Cairo, Egypt;; b School of Pharmacy, University of Waterloo, ON, Canada

**Keywords:** Nasal route, direct nose to brain pathway, *in situ* gel, absolute bioavailability

## Abstract

Agomelatine (AGM) is an antidepressant drug with a low absolute bioavailability due to the hepatic first pass metabolism. AGM-loaded solid lipid nanoparticles were formulated in the form of an *in situ* gel to prolong the intranasal retention time and subsequently to increase the absorbed amount of AGM. The optimized *in situ* gel formula had a sol–gel transition temperature of 31 °C ± 1.40, mucociliary transport time of 27 min ±1.41%, released after 1 and 8 h of 46.3% ± 0.85 and 70.90% ± 1.48. The pharmacokinetic study of the optimized *in situ* gel revealed a significant increase in the peak plasma concentration, area under plasma concentration versus time curve and absolute bioavailability compared to that of the oral suspension of Valdoxan® with the values of 247 ± 64.40 ng/mL, 6677.41 ± 1996 ng.min/mL, and 37.89%, respectively. It also gave drug targeting efficiency index of 141.42 which revealed more successful brain targeting by the intranasal route compared to the intravenous route and it had direct transport percent index of 29.29 which indicated a significant contribution of the direct nose to brain pathway in the brain drug delivery.

## Introduction

Agomelatine (AGM) is a novel antidepressant. Pharmacodynamically being a melatonin analog, it has a potent agonistic action on melatonin MT1 and MT2 receptors (Millan et al., 2003). In addition, it has serotonin 5-HT2C receptor antagonistic activity (Yous et al., 1992; Audinot et al., 2003; Millan et al., 2003).

Pharmacokinetically, AGM is well absorbed (> 75%) after oral administration but it is subjected to extensive first pass metabolism leading low absolute bioavailability (< 5%) (Zupancic & Guilleminault, 2006). The study seeks to achieve two goals, firstly decreasing first pass metabolism of AGM and therefore enhancing its absolute bioavailability and secondly increasing AGM brain delivery.

To achieve these targets, we prepared mucoadheive thermosensitive *in situ* gel conatining AGM loaded SLNs given by the intranasal route. The intranasal route was selected firstly as drugs are absorbed from the nasal cavity away from the portal circulation which is expected to enhance absolute bioavailability and secondly to make use of the direct pathways between the nose and brain through which AGM can be delivered to brain without the necessity of crossing the BBB which is expected to enhance the brain bioavailability. The olfactory region of the nasal epithelium and the trigeminal neural region are two direct nose to brain pathways (Alsarra et al., 2010; Haque et al., 2014). The olfactory epithelium, which represents about 5% of the total area of the nasal cavity in man (Soane et al., 2001), is a pseudostratified epithelium containing the olfactory nerves through which AGM can reach the CNS via extracellular or intracellular transport (Dhuria et al., 2010). In addition AGM can use the trigeminal nerve branches innervating the respiratory and olfactory epithelium of the nasal cavity to enter the CNS (Kozlovskaya et al., 2014).

Besides the direct pathways to reach brain, there is an indirect pathway in which AGM is absorbed from nasal cavity into systematic circulation and then permeates through the BBB to reach brain. To enhance the permeation of AGM through the BBB and its ability to reach brain using the indirect pathway, we previously loaded it into solid lipid nanoparticles (SLNs) (Fatouh et al., 2017) which are thought to enhance the BBB permeation due to their endocytosis by the BBB endothelial cells, their inhibitory action on the transmembrane efflux systems or the solubilizing action of the surfactant associated with their preparation (Gastaldi et al., 2014). SLNs are colloidal particles having size from 1 nm to 1000 nm prepared using lipids with melting points higher than the room temperature (Battaglia & Gallarate, 2012; Lasa-Saracibar et al., 2012; Gastaldi et al., 2014).

Due to the low viscosity and lack of mucoadhesivness, dispersion of AGM loaded SLNs is expected to have short nasal residence time which may decrease both nasal systemic absorption and direct nasal brain transport. Therefore, SLNs dispersion was formulated in the form of an *in situ* gel which remains as a liquid dosage form outside the body and undergoes sol–gel transition when it comes in contact with the body fluids. *In situ* gel appears as a promising intranasal dosage form as it possess, like the liquid dosage forms, the advantage of the easy and accurate administration and simultaneously the advantages of the limited nasal clearance, the prolonged nasal retention time, and the sustained release profile like the solid and semisolid dosage forms (Illum et al., 1994; Watts & Smith, 2009). In our study, we prepared temperature induced *in situ* gel system which converts into the gel form due to the relatively elevated temperature of the body (Huffman et al., 1986, Nanjawade et al., 2007). Pluronic F 127, which is triblock copolymer composed of polyethylene oxide (PEO) and polypropylene oxide (PPO) units (Ruel-Gariepy & Leroux, 2004), was used as thermosensitive polymer in the *in situ* gel formulation. To impart mucoadhesive properties and increase the nasal retention time of the *in situ* formulation, a mucoadhesive polymer was also used.

## Materials and methods

### Materials

Agomelatine was kindly provided by HikmaPharma (Cairo, Egypt), Polyvinylalcohol (Mwt 22,000), sodium deoxycholate, Pluronic F 127, Carbopol, Chitosan [low molecular weight (30–200 cps)], sodium carboxymethylcellulose (Na CMC), sodium alginate, and Hydroxypropylmethylcellulose (HPMC) (1500–4500 cps) were purchased from Sigma Chemical Co. (St. Louis, MO). Gelucire 43/01 were kindly provided by Gattefosse Co. (Saint-Priest, France). Dichloromethane was purchased from El-Nasr Pharmaceutical Chemicals Co. (Cairo, Egypt). All other reagents were of analytical grade.

### *Preparation of* SLNs

Solid lipid nanoparticles were prepared in our laboratory according to the procedures outlined by Fatouh et al. (2017). The obtained emulsion was subjected to ultra-sonication using probe sonicator (Ultrasonic processor model VCX 750) at the amplitude of 40 W for 5 minutes to decrease the globules size to the required nanometer range. The final step was stirring the prepared emulsion at room temperature using magnetic stirrer at 400 rpm to allow the organic solvent to evaporate and SLNs to be formed.

### *In vitro characterization of* SLNs

#### Determination of particle size (PS), polydispersity index (PDI), and zeta potential (ZP)

The mean PS, PDI, and ZP were determined by dynamic light scattering (DLS, Zetasizer Nano ZS, Malvern Instruments, Malvern, UK) at 25 °C with 90° measurment angle. Before measurement, the formulation was diluted with distilled water in the ratio of 1–20 to have a suitable scattering intensity. Each sample was measured twice and the data are provided as mean ± standard deviation.

#### Determination of the entrapment efficiency (EE%)

The EE% of AGM loaded SLNs was determined by measuring the concentration of the free drug in the aqueous phase of the SLNs dispersion. A definite volume of the prepared SLNs dispersion was diluted with distilled water in the ratio of 1–20 and centrifuged using Cooling centrifuge (Beckman, Fullerton, Canada) at 15,000 rpm for 1 h at 4 °C. The unentrapped drug concentration was estimated spectrophotometrically at 276.4 nm using UV/VIS spectrophotometer (Shimadzu, Kyoto, Japan). The EE% was calculated using the following equation

The EE%=Winitial-WfreeWinitial × 100

### Preparation of in situ gels

*In situ* gels were prepared using the cold method described by Schmolka et al. (Schmolka, 1972; Zaki et al., 2007). Briefly a calculated amount of a mucoadhesive polymer was dissolved in a definite volume of SLNs dispersion. Pluronic F 127 was added slowly to the dispersion with continuous stirring on a magnetic stirrer. The dispersion was then stored in a refrigerator until clear solution was obtained. Drop wise addition of 0.1 N acetic acid was needed to dissolve Chitosan.

### Preliminary study for the optimum conditions

During the preliminary trials, different concentrations of Pluronic F 127 and types of mucoadhesive polymers were used to prepare the *in situ* gels. The used concentrations of Pluronic F 127 were 16, 17, 18, 19, and 20% w/v. The used types of nucoadhesive polymers were Chitosan, Carbopol, Na alginate, Xanthan gum, Na carboxymethylcellulose, and HPMC. The mucoadhesive polymers were used in concentration of 0.2% w/v. One-way ANOVA with subsequent LSD test was performed to compare the sol–gel transition temperatures of the formulations using SPSS 17^®^ software.

### Optimization of the in situ gels using a full factorial design

Based on the results of the preliminary trials and using Design-Expert^®^ software, a 2^2^ × 3^1^ full factorial design was constructed based on three independent variables namely: concentration of Pluronic F 127 (X_1_), type of mucoadhesive polymer (X_2_), and concentration of mucoadhesive polymer (X_3_). Pluronic F 127 was used in two concentrations 16 and 18% (w/v). Two types of mucoadhesive polymers (HPMC and Na alginate) were used. Each mucoadhesive polymer was used in 3 concentrations 0.2, 0.4, and 0.6% (w/v). The dependent variables measured were T sol to gel (Y_1_), flow index (*n*) (Y_2_), mucociliary transit time (Y_3_), the % released of AGM after 1 hour ‘Q1h’ (Y_4_), the % released of AGM after 8 h ‘Q8h’ (Y_5_), and particle size ‘PS’ (Y_6_).

### In vitro and in vivo characterization of in situ gel

#### Measurement of the sol–gel transition temperatures

The sol to gel transition temperature was detected as described by Gilbert et al. (1987) and Vadnere et al. (1984). Briefly, 2 mL aliquot of the prepared solution was transferred to a test tube which was covered by a parafilm and fixed in a digital circulating water bath. The temperature of the bath was increased from 20 to 40 °C in increments of 0.5 °C. With each temperature increment, the test tube was allowed to equilibrate with the water bath temperature for 10 minutes then tested for gelation by tilting it 90°.

#### Measurement of steady shear viscosity

The rheological properties of the *in situ* gels were evaluated using cone and plate viscometer (Brookfield viscometer; type DVT-2). The temperature of the plate was fixed at 35 ± 0.1 °C by connecting it to a thermostatic water bath. A sample of the sol was added to the plate using a spatula. The rate of shear was increased gradually and viscosity was determined from the instrument readings. The results were fitted to the power law constitutive equation (Tung, 1994): η = my*^n^* ^−1^.

From the equation, the consistency index (m) and the flow index (n) for each formula were obtained. The consistency index parameter gives an idea of the viscosity of the fluid while the flow index gives indication of the flow type. If *n* = 1 this indicates Newtonian behavior while if it is less than 1, this corresponds to shear thinning flow. As the value of *n* decreases lower than 1, the shear thinning behavior increases.

#### *In vitro release of* AGM *from prepared in situ gels*

The *in vitro* release study was carried out by the dialysis bag diffusion technique. The formulations were stored in refrigerator overnight to be in the sol state. A one milliliter aliquot of each formulation was filled into dialysis bag [molecular weight cutoff 12,000–14,000 Da, Sigma Chemical Co. (St Louis, MO)]. The filled dialysis bag was immersed in a bottle containing 100 mL phosophate buffer pH 6.8. The release was carried out using a thermostatic horizontal shaker (GFL, Gesellschatt laboratories, Berlin, Germany) established at 100 rpm and 35 ± 0.5 °C. Aliquots were withdrawn at 15 min, 30 min, 1, 2, 4, 6, and 8 h time intervals. The drug concentration was measured spectrophotometrically at 276.4 nm using UV/VIS spectrophotometer (Shimadzu, Kyoto, Japan). Experiments were repeated three times and the results were expressed as the mean values ± standard deviation (SD).

#### Nasal mucociliary transport time

The *in vivo* nasal mucociliary transport time was determined by a method reported by Lale et al. (1998) with modification. Briefly, rats were anesthetized by intramuscular injection of sodium thiopental (7 mg/kg). A 10 μl of each *in situ* gel containing methylene blue dye (5 mg/mL) was instilled to a rat nose (5 mm depth into the right nostril) using a micropipette. The appearance time of the blue dye at nasopalatine and pharynx was detected by swabbing these regions in oral cavity with moistened cotton-tipped applicators, at every minute after dosing for 60 min. The appearance time of the blue dye was recorded. Methylene blue was dissolved in normal saline at the same concentration (5 mg/mL) and was used as the control.

#### Particle size

To study the effect of incorporating the SLNs into *in situ* gel, the mean PS of the SLNs in the *in situ* gel formulae was determined by dynamic light scattering (DLS, Zetasizer Nano ZS, Malvern Instruments, Malvern, UK) at 25 °C. Before measurement, the formulation was properly diluted with distilled water to have a suitable scattering intensity.

### Elucidation of optimized formula

Numerical optimization was performed using Design Expert^®^ software to maximize mucociliary transit time and Q8h, minimize flow index and Q1 h and have sol to gel transition temperature in the range of 25–33 °C. The simultaneous optimization technique described by Derringer and Suich (1980) was chosen for optimization of the responses. This method is based on the utilization of desirability functions. Each response is converted into an individual desirability function di that can be varied over the range 0 < di <1. The design variables are then selected to maximize the overall desirability adopting the following equation.

$$D={\left(d_{1}d_{2}d_{3},\ldots dm\right)}^{1/m}$$

where D is the overall desirability, di is the individual desirability and m is the number of responses to be optimized.

### Transmission electron microscopy

The morphology of the selected *in situ* gel formula was examined by the transmission electron microscope (JEM-1010; JEOL Ltd., Tokyo, Japan). One drop of diluted *in situ* gel was deposited on a carbon-coated copper grid and negatively stained by 2% (w/v) aqueous phosphotungstic acid solution and then examined at 80 kV.

### Pharmacokinetic study

#### Administration of AGM to rats

The study, whose protocol was reviewed and approved (PI 1197) by the institutional review board; Research Ethics Committee-Faculty of Pharmacy, Cairo University (REC-FOPCU), involved 72 male rats (weight 70–250 g). The rats were divided into three groups where group 1 received *in situ* gel (Gel-3) through the intranasal route, group 2 received aqueous solution of AGM in normal saline containing 10% ethanol through the intravenous route, and group 3 received oral suspension of Valdoxan^®^. All animals received AGM in a dose of 2.14 μg/g which was calculated based on the FDA guidelines in the conversion of animal doses to human equivalent doses (UFaDA, 2005). At different time intervals 2, 5, 10, 15, 30, 60, 120, 180, and 360 minutes following the administration of AGM formulations, three rats of each group were sacrificed. Blood was collected from the trunk and put into heparinized tubes, centrifuged at 4000 rpm for 15 min at 25 °C and plasma separated. Brain tissue samples were taken after cutting the skulls followed by homogenization using ultra turrax homogenizer with three-folds volumes of saline at 24,000 rpm for 1 min. Homogenized brain and separated plasma tubes were stored at –80 °C until being assayed.

#### Assay of AGM in plasma and brain

AGM was analyzed in plasma and homogenized brain samples using an Applied Biosystems/MDS Sciex liquid chromatography tandem mass spectrometry (LC-MS/MS). Plasma or homogenized brain samples (0.5 mL) were placed in 7 mL glass tubes, and then 50 μL of internal standard solution (100 ng/mL Clonazepam) was added. Samples were then vortexed for 1 min. The extraction solvent [4 mL tertiary butylmethyl ether (TBME)] was added; the tubes were then mixed for 10 min on a rocker-mixer Reax II (Heidolph, Schwabach, Germany). Samples were centrifuged at 1790*g* for 10 min at 4 °C, using Eppendorf centrifuges 5804 R and the upper organic layer was transferred into new Wassermann tubes and evaporated to dryness using vacuum concentrator (Eppendorf 5301; Hamburg, Germany). Dry residues were reconstituted by the addition of 0.25 mL of mobile phase, and then tubes were vortex mixed for 1 min and finally placed into the auto-sampler for LC-MS/MS analysis. An aliquot of 10 μL of the samples was injected into a Shimadzu Prominence (Shimadzu, Japan) series LC system equipped with degasser (DGU-20A3) using Agilent C18 column (Agilent) (50 × 4.6 mm) with 3.5 mm particle size. The isocratic mobile phase (80% acetonitrile +20% water containing 0.1% formic acid) was delivered at a flow rate of 1.0 mL/min into the mass spectrometer’s electrospray ionization chamber. Quantitation was achieved by MS/MS detection in positive ion mode for both AGM and clonazepam IS using a MDS Sciex (Foster City, CA) API-3200 mass spectrometer, equipped with a Turbo Ionspray interface at 500 °C. The ion spray voltage was set at 5500 V. The common parameters, viz., nebulizer gas, curtain gas, auxiliary gas, and collision gas were set at 25, 20, 40, and 6 psi, respectively. The compound parameters, namely, declustering potential (DP), collision energy (CE), entrance potential (EP), and collision exit potential (CXP) were 41, 6.5, 25, 4 V for AGM and 51, 9.5, 31, 6 V for clonazepam (IS), respectively. Detection of the ions was performed in the multiple reactions monitoring (MRM) mode, monitoring the transition of the m/z 244.03 precursor ion to the m/z 185.30 for AGM and m/z 315.96 precursor ion to the m/z 270.00 for IS. The Q1 and Q3 quadrupoles were set on unit resolution. The analytical data were processed by Analyst software (version 1.4.2).

#### Pharmacokinetic analysis

The mean concentrations of AGM in plasma and brain samples were plotted against time and the peak plasma and brain concentrations (*C*_max_) as well as the time to reach these peaks (tmax) were read directly. The area under AGM concentration-time curve (AUC _0–360 min_) was calculated by the trapezoidal method without extrapolation to infinity. The time to reach half plasma concentration (*t*_1/2_), and the mean residence time (MRT) were calculated using Kinetica software program (version 4.4.1). The absolute bioavailability (AB) of the intranasal formulations compared to IV solution was calculated.

To evaluate AGM brain targeting after nasal dosing, two indices were calculated.

(1) Drug targeting efficiency (DTE%) which compares the delivery of drug to brain following intranasal administration versus systemic administration and calculated as follows:

$$DTE\%\;=\frac{\left[\frac{AUC\;brain\;0–t}{AUC\;plasma\;0–t}\right]IN}{\left[\frac{AUC\;brain\;0–t}{AUC\;plasma\;0–t}\right]IV}\times 100$$

The value of %DTE can range from –∞ to ∞, and the values higher than 100% indicate a superior drug delivery to the brain following intranasal administration as compared to the systemic administration.

(2) Nose-to-brain direct transport percentage (DTP%) which measures the relative contribution of the direct nose to brain routes in the overall delivery to the brain (i.e. via the direct routes and via the BBB) is calculated as follows:

$$DTP\%=\frac{B\;IN\;-BX}{B\;IN}\times 100$$

where BIN is AUC_0–t_ (brain) following IN administration and BX is the brain AUC fraction contributed by systemic circulation through the BBB following intranasal administration, and equals: $\frac{B\;IV}{P\;IV}\times \;PIN\;$ where BIV is AUC_0–t_ of AGM in the brain following IV administration, PIV is AUC_0–t_ of AGM in the plasma following IV administration and PIN is AUC_0–t_ of AGM in the plasma following intranasal instillation.

The value of %DTP can range from –∞ to 100%, the negative values indicate more efficient drug delivery to brain through the BBB permeation than the direct nose to brain routes while the positive values of the %DTE indicate a significant contribution of the direct routes to the overall brain delivery.

## Results and discussion

In order to prolong nasal retention time of AGM and increase its nasal absorption, selected SLNs from our previous work (Fatouh et al., 2017) was formulated as *in situ* gel. The selected SLNs formula had particle size of 167.7 nm ±0.42, zeta potential of –17.9 mV ±2.7, polydispersity index of 0.1285 ± 0.1, entrapment efficiency % of 91.25% ± 1.7, Q1h and Q8h of 35.4% ±1.13 and 80.87% ± 5.16, respectively. This formula was prepared using 3% w/v of Gelucire 43/01, 0.5% w/v of Na deoxycholate, 1% w/v of PVA and 0.4% w/v of AGM. Pluronic F 127 was used as a base for preparing thermosensitive *in situ* gel formulation. During the preliminary trials, Pluronic F 127 was dissolved in the SLNs dispersion in concentrations of 16, 17, 18, 19, and 20% w/v. According to the one-way ANOVA test, there is a significant difference in T sol–gel between each pair of the concentrations (*p* < .05). An inverse relationship between the concentration of pluronic F 127 and the T sol–gel was noticed. To reinforce gelation strength and mucoadhesive properties of the solutions, a mucoadhesive polymer was added to the formulation. Different types of nucoadhesive polymers (chitosan, carbopol, Na alginate, xanthan gum, Na CMC, and HPMC) were tried in the *in situ* gel formulation in concentration of 0.2% w/v.

As illustrated in Table 1, except for Na alginate the incorporation of a mucoadhesive polymer caused a significant decrease in the T sol–gel which agreed with Dias et al. (2010). The T sol–gel depression can be attributed to the hydration of the mucoadhesive polymer which consumes a part of the formulation water content causing Pluronic F 127 micelles to be less hydrated. Therefore, the micelles dehydration and the sol to gel transition occurred at lower temperature (Sharma et al., 2008). The optimum T sol–gel of a thermosensitive *in situ* gel for intranasal administration is between 25 and 32 °C so that it remains in the liquid state at room temperature to ensure the ease and accuracy of administration but sets into gel at the nasal temperature (32–35 °C) to increase the contact time with the nasal mucosa leading to sustained and controlled release. From the results, the mucoadhesive polymers that did not change the gelation temperature outside the acceptable range for the nasal administration are Na alginate and HPMC. So they were selected for the further trials. As shown in Table 2, using Design-Expert^®^ software, a 2^2^ × 3^1^ full factorial design was constructed to evaluate the effect of the concentration of Pluronic F 127 (X_1_), the type of mucoadhesive polymer (X_2_), and the concentration of mucoadhesive polymer (X_3_) on the T sol to gel (Y_1_), consistency index (m) (Y_2_), flow index (n) (Y3), Q 1 h (%)(Y_4_), Q 8 h (%)(Y_5_) and mucociliary transit time (Y_6_), The experimental trials were performed at all 12 possible combinations.

**Table 1.** The sol–gel transition temperature of agomelatine loaded SLNs *in situ* gels prepared using 18% w/v of Pluronic F 127 and 0.2% w/v of different types of mucoadhesive polymers.

Mucoadhesive polymer	Sol–gel transition temperatures (°C)
Carbopol	22.25 ± 1.06
Chitosan	20.50 ± 0.71
Na alginate	31.50 ± 2.12
Na CMC	22.50 ± 2.12
HPMC	29.00 ± 1.41

**Table 2.** Experimental runs, independent variables and measured responses of the full factorial experimental design of agomelatine-loaded SLNs *in situ* gels.

Formula	X_1_: Pluronic conc.	X_2_: type of mucoadhesive polymer	X_3_: conc. of mucoadhesive polymer	Y_1_: gelation temp.	Y_2_: m (consistency index)	Y_3_: n (flow index)	Y_4_: Q 1 h	Y_5_: Q 8 h	Y_6_: Mucociliary transport time	Y_7_: PS
G1	16%	HPMC	0.2%	40.00 ± 1.40	1402.50	0.82	15.14 ± 0.56	56.50 ± 0.71	10.50 ± 0.71	171.25 ± 1.76
G2		Na alginate		43.00 ± 0.71	903.30	0.98	11.04 ± 0.39	62.50 ± 0.49	6.50 ± 2.10	183.00 ± 0.71
G3		HPMC	0.4%	31.00 ± 1.40	43,762.20	0.20	15.18 ± 0.67	46.30 ± 0.85	27.00 ± 1.41	175.75 ± 1.10
G4		Na alginate		42.00 ± 0.71	700.70	0.96	14.01 ± 0.31	57.75 ± 0.38	6.00 ± 1.41	191.10 ± 6.50
G5		HPMC	0.6%	27.00 ± 2.10	30,902.90	0.021	14.26 ± 0.62	54.80 ± 0.78	24.00 ± 0.71	185.50 ± 1.69
G6		Na alginate		39.00 ± 0.71	529.50	0.95	13.56 ± 0.61	69.12 ± 0.76	4.5.00 ± 0.71	183.25 ± 4.59
G7	18%	HPMC	0.2%	27.00 ± 1.41	25,703.90	0.13	16.99 ± 0.84	54.20 ± 1.06	28.00 ± 1.41	193.40 ± 4.80
G8		Na alginate		35.00 ± 2.80	1338.30	0.45	10.28 ± 0.63	59.37 ± 0.79	8.50 ± 0.71	176.40 ± 5.09
G9		HPMC	0.4%	24.00 ± 0.35	43,431.00	0.16	21.20 ± 1.18	54.40 ± 1.48	26.00 ± 1.41	189.10 ± 8.34
G10		Na alginate		33.00 ± 1.41	4532.70	0.38	12.38 ± 1.06	68.12 ± 1.32	9.00 ± 2.80	177.90 ± 1.55
G11		HPMC	0.6%	23.00 ± 2.12	46,773.50	0.16	8.94 ± 1.35	37.60 ± 1.69	28.50 ± 0.71	182.70 ± 3.25
G12		Na alginate		33.00 ± 1.40	5937.50	0.32	19.14 ± 1.62	57.12 ± 2.03	10.50 ± 2.10	179.25 ± 1.06

### Effect of the formulation variables on the gelation temperature of in situ gel

The T sol–gel of the design *in situ* gel formulae ranged from 23 to 43 °C as shown in Table 2. The formula giving the lowest T sol–gel is G-11 while the formula giving the highest T sol–gel is G-2. According to the ANOVA test results, the concentration of Pluronic F 127 (X_1_) and the type of the mucoadhesive polymer (X_2_) had significant effects on the T sol–gel. It was observed that the formulae containing 18% w/v had significantly lower T sol–gel than that of the formulae containing 16% w/v (*p* = .0215). The inverse relationship between the concentration of Pluronic 127 and the T sol–gel agreed with previous reports (Cabana et al., 1997; Desai et al., 2001; Matthew et al., 2002). The sol–gel transition of Pluronic F 127 dispersions depends on the dehydration of Pluronic F 127 micelles cores with the temperature rise. With high concentration of Pluronic F 127, the number of micelles formed increased with small water content expected to hydrate P 127 chains. Therefore the micelles dehydration and the sol to gel transition occurred at lower temperature. For the type of the mucoadhesive polymer (X_2_), it was observed that the formulae containing HPMC had significantly lower sol to gel transition temperature than that of the formulae containing Na alginate (*p* = .0170) which can be explained based on the larger molecular weight of HPMC which expected to bind to more water molecules causing the dehydration of Pluronic F 127 micelles at lower T sol–gel.

### Effect of the formulation variables on the consistency index and the flow index

The consistency index of the design *in situ* gel formulae ranged from 529.5 to 46,773.5 as shown in Table 1. The formula giving the lowest consistency index is G-6 while the formula giving the highest consistency index is G-11. According to the ANOVA test results, the only factor having significant effect on the consistency index is the type of the mucoadhesive polymer (X2) where the formulae containing HPMC have significantly higher consistency index than that of the formulae containing Na alginate (*p* = .0181). This can be explained based on the T sol–gel of the two polymers. During rheological properties evaluation, the viscometer cone was fixed at 35 ± 0.1 °C which is sufficient for sol–gel transition in case of all the formulae containing HPMC except G1 leading to higher consistency index. On the other hand, this temperature is lower than sol–gel transition temperature of the formulae G2, G4, and G6 prepared using Na alginate therefore these Na alginate containing formulae remained in the sol state giving low values of the consistency index.

The flow index of the design *in situ* gel formulae ranged from 0.021 to 0.98 (Table 2). The formula giving the lowest flow index is G-3 while the formula giving the highest flow index is G-2. According to the ANOVA test results, no factor has a significant effect on the flow index. The flow index results can be explained based on T sol–gel of the prepared formulae. It was found that the formulae, having sol–gel transition temperature lower than or equal to 35 °C (G-3, G-5, G-7, G-8, G-9, G-10, G-11, and G-12) have been transformed into the gel form during the evaluation of the rheological properties leading to a non-Newtonian shear thinning rheological behavior with small flow index (0.0215–0.45). On the other hand the formulae, having sol–gel transition temperature higher than 35 °C (G-1, G-2, G-4, and G-6) remained in the sol state leading to a Newtonian rheological behavior with large flow index (0.82–0.9843).

### Effect of the formulation variables on the amount released after 1 h (Q1h) and the amount released after 8 h (Q8h)

**Figure 1.**
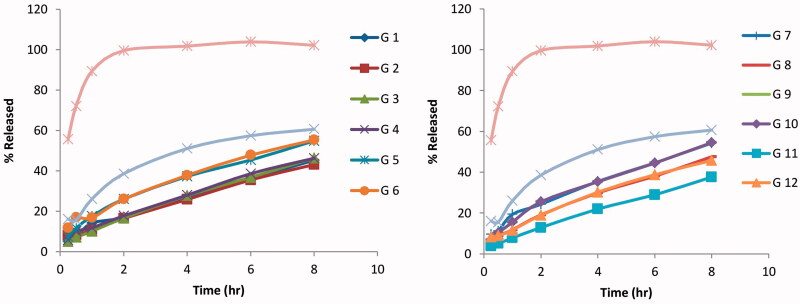
Release profiles of the full factorial design agomelatine loaded solid lipid nanoparticles *in situ* gel formulae (A) G1–G6) and (B) G7–G12 in phosphate buffer (pH 6.8) and at 37 °C.

Compared to the release profile of the free AGM solution and the optimized AGM loaded SLNs formula as shown in Figure 1, significant reduction in the release rate was achieved in the *in situ* gel formulae (*p* < .05). The % released of AGM after one hour (Q1h) was 89.35% for the free AGM solution and 35.40% for SLNs dispersion while it ranged from 8.94 to 21.2% for the *in situ* gel formulae. The formula giving the lowest Q1h is G-11 while the formula giving the highest Q1h was G-9. On the other hand, the % released of AGM after 8 h (Q8h) was 102.18% for the free AGM solution and 80.87% for SLNs dispersion while it ranged from 37.60 to 69.12% for the *in situ* gel formulae. The formula giving the lowest Q8h is G-11 while the formula giving the highest Q8h was G-6. In the comparison between the release profiles of the full factorial design AGM loaded SLNs *in situ* gel formulae and according to ANOVA test results, no factor has a significant effect on Q1h while the type of the mucoadhesive polymer (X2) has a significant effect on Q8h. The formulae containing HPMC have significantly lower Q8h than that of the formulae containing Na alginate (*p* = .0055). This can be explained firstly based on the sol–gel transition where most of HPMC containing formulae were transformed from sol to gel state at the temperature at which the *in vitro* release was carried out leading to slower release profile while the half of Na alginate containing formulae remained in sol state leading to faster release profile and higher Q8h. Secondly because of the higher consistency index of HPMC containing formulae, the drug diffusion occurred at slower rate.

### Effect of the formulation variables on the mucociliary transport time

The mucociliary transport time of the factorial design *in situ* gel formulae ranged from 4.5 to 28.5 minutes (Table 2). The formula giving the shortest mucociliary transport time is G-6 while the formula giving the longest mucociliary transport time is G-11. According to ANOVA test results, the only factor having significant effect on the mucociliary transport time was the type of the mucoadhesive polymer (X2) where the formulae containing HPMC have significantly higher mucociliary transport time than that of the formulae containing Na alginate (*p* = .0328). This can be explained by the success of sol–gel transition after nasal administration of the HPMC containing formulae and also by the higher consistency index of these formulae leading to longer intranasal retention time and mucociliary transport time. It can be concluded that the mucoadhesion force of HPMC containing formulae was higher than that of Na alginate containing formulae. According to ANOVA test done using SPSS® software, all the *in situ* gel formulae gave significantly longer mucociliary transport time that the control that had mucociliary transport time of 3.5 min ±0.71 (*p* < .05).

### Effect of the formulation variables on the PS of the in situ gels

The particle size of the SLNs in the factorial design *in situ* gel formulae ranged from 171.25 to 193.4 nm as shown in Table 2. According to the ANOVA test results, there was no factor having significant effect on the PS (*p* > .05). There is no significant difference in the PS of the SLNs in the *in situ* gel formulae compared to the optimized SLNs formula. Therefore it can be concluded that the *in situ* gel formulation did not affect the PS of the SLNs.

### Optimization of in situ gel formulae

For selection of the optimum formula, the optimization criteria were set to minimize flow index and Q1h, maximize Q8h and mucociliary transport time and to have T sol–gel in the range of 25–33 ***°*** C. The formula giving the highest numerical desirability is G-3 with a value of 0.65. The predicted responses for this formula are T sol–gel of 31.58 ***°*** C, consistency index of 40,207.60, and flow index of 0.23, Q1h of 15.10%, Q8h of 46.28% and mucociliary transport time of 25.16 minutes while the observed responses are T sol–gel of 31 ± 1.4 ***°*** C, consistency index of 43,762.2, flow index of 0.2004, Q1h of 15.18%** **± 0.85, Q8h of 70.9%** **± 1.48 and mucociliary transport time of 27 ± 1.41 minutes. Results revealed high similarity between the observed and predicted values of the optimal formula with bias % did not exceed 12.80%. Hence, G-3 can be considered as a promising *in situ* gel formula; therefore it was selected for further investigations.

### Morphology of in situ gel

**Figure 2.**
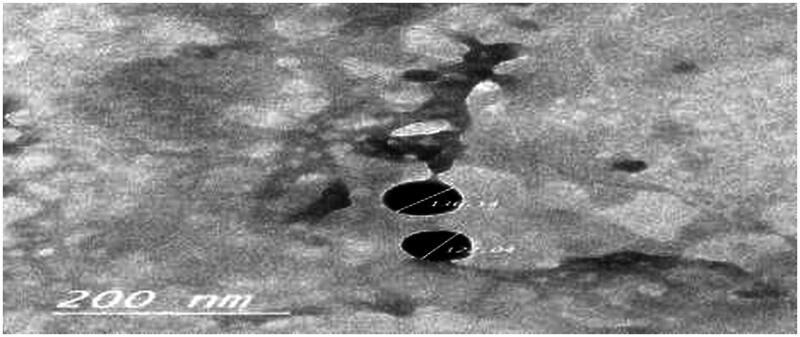
Transmission-electron micrograph (TEM) of the optimized agomelatine loaded solid lipid nanoparticles *in situ* gel formula.

The TEM micrograph of the selected *In situ* gel formula (G-3) as shown in Figure 2 demonstrated that the nanoparticles are well identified and present in a nearly perfect spherical shape and the particle size was close to that determined by the dynamic light scattering.

### Pharmacokinetic study

The liquid chromatography–mass spectrometry assay has a good linearity from 0.01 to 100 ng/mL with acceptable interday accuracy ranged from 93.2 to 105.1%, while the interday precision ranged from 3.1 to 9.7%. The accuracy of freeze and thaw stability ranged from 87.6 to 95.7%, while its precision ranged from 4.8 to 8.9%.

**Figure 3.**
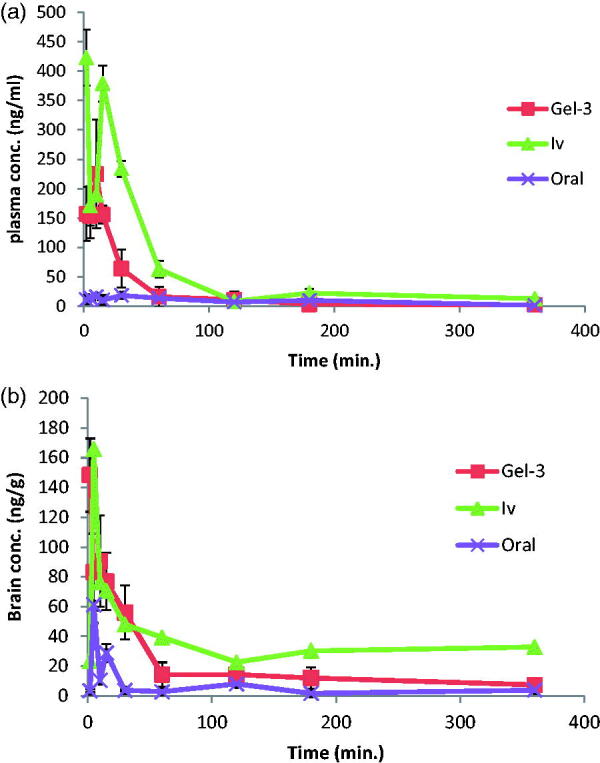
Agomelatine (A) mean plasma concentrations and (B) mean brain concentrations after administration of intranasal Gel-3, IV agomelatine solution, and oral agomelatine suspension.

Figure 3(A) and 3(B) denotes the mean AGM concentrations in plasma and brain of rats after administration of intranasal Gel-3 (G-3), IV AGM solution and oral Valdoxan® suspension. As shown in Table 3, G-3 was found to have significantly higher Cmax, AUC (0-360 min.) and absolute bioavailability 247.00 ng/mL, 6677.41 ng min/mL, and 37.89%, respectively than that of the oral suspension of Valdoxan® (20.73 ng/mL, 2828.08 ng min/mL, and 16.12%, respectively) (*p* < .0001). The observed increase in *C*_max_, AUC 0–360 min, and absolute bioavailability of the intranasal G-3 can be explained as follow: first, the systemic absorption of AGM from the intranasal G-3 is carried out directly into the systemic circulation avoiding the hepatic first pass metabolism causing increased amount of AGM to reach the systemic circulation. Second, the lipophilic nature of SLNs being composed of lipids is expected to enhance the ability of the particles to partition into the lipid bilayer of the nasal epithelial cell membrane and pass directly through the cells showing higher systemic absorption. Third, the used cosurfactant SDC is known to enhance the nasal absorption via several possible mechanisms including enhancing permeability of the membrane structure through opening of tight junctions between epithelial cells (Behl et al., 1998).

**Table 3.** Pharmacokinetic parameters of agomelatine in both plasma and brain.

		Rabbits plasma			Rabbits brain	
Parameters	Gel-3	IV	Oral	Gel-3	IV	Oral
Cmax (ng/mL)	247.00 ± 64.40	423.00 ± 47.90	20.70 ± 3.19	148.33 ± 64.36	165.60 ± 37.70	61.20 ± 12.23
Tmax (min)	7.33 ± 4.62	2.00 ± 0.00	15.67 ± 14	2.00 ± 4.61	5.00 ± 0.00	5.00 ± 0.00
AUC_0–_ *_*t*_* (ng/mL min)	6677.41 ± 1996.00	17,616.00 ± 2839.00	2828.10 ± 969.00	6570.23 ± 1995.50	12,499.60 ± 4166.50	1710.12 ± 378.24
K (min^−1^)	0.0041 ± 0.0004	0.0032 ± 0.0003	0.0079 ± 0.002			
*t*_1/2_ (min)	378.93 ± 365.00	216.74 ± 23.80	91.98 ± 23.80			
MRT (min)	225.28 ± 227.00	173.23 ± 5.59	151.64 ± 28.00	182.70 ± 227.07	163.19 ± 6.11	8206.90 ± 5505.04
Absolute bioavailability	37.89		16.12			
Relative bioavailability	234.98					
Drug targeting efficiency (DTE)	141.42					
Direct transport percentage (DTP)	29.29					

G-3 had also a significantly longer half-life (378.92 min) than the oral suspension of Valdoxan® (91.98 min.) (*p* < .0001) which can be explained based on that most of AGM in G-3 was entrapped (EE% = 91.25%) so it was released and subsequently eliminated at a slower rate. Also as having particle size <200 nm, SLNs could bypass the filtration at the interendothelial slits (IES) in the walls of venous sinuses and subsequently had reduced reticuloendothelial clearance and prolonged circulation time (Moghimi et al., 1991; Gastaldi et al., 2014). In addition, the presence of PVA on the surface of the particles decreased their reticuloendothelial clearance and prolonged their blood circulation time.

G-3 had also significantly lower tmax (7.33 min) than the oral suspension of Valdoxan® (15.67 min) (*p* = .001) which can be attributed to the faster systemic absorption from the nasal route than the oral administration.

Figure 3(B) denotes the mean AGM concentrations in brain of rats after administration of intranasal G-3, IV AGM solution and oral Valdoxan® suspension. As shown in Table 3, G-3 were found to have significantly higher *C*_max_ and AUC (0–360 min) (148.33 ng/mL and 6570.23 ng min/mL, respectively) than that of the oral suspension (61.20 ng/mL and 1710.12 ng min/mL, respectively) (*p* < .0001). The enhanced brain drug delivery can be explained based on several factors. Firstly, the enhanced absolute bioavailability and the higher AGM plasma concentrations with the intranasal G-3 could create a higher driving force for the diffusion across the BBB due to the increased concentration gradient from the systemic circulation to the brain. Secondly, drug loaded SLNs had preferential BBB permeation compared to the free dug form. The enhanced BBB permeation of the SLNs could be related to the lipid content of the SLNs which could enhance the transcellular diffusion across the BBB. Also, the surfactants associated with the SLNs like SDC and PVA acted as absorption enhancers, decreased nanoparticle clearance by the reticuloendothelial system (RES), and inhibited the efflux system, especially P-glycoprotein (Pgp) enhancing the transport across BBB (Esposito et al., 2008; Tsai et al., 2012). The associated surfactants could also increase brain uptake via transient opening of the brain endothelial tight junctions (Alam et al., 2010). The SLNs could also be endocytosed by the BBB endothelial cells (Kreuter, 2005) from where they were transcytosed into the brain (Dehouck et al., 1997) or released AGM within the endothelial cells that diffused into the brain (Kreuter, 2005). Thirdly, the preferential brain delivery of the SLNs can be attributed to the direct transport from the nose to brain where AGM loaded SLNs or the free AGM could reach the brain using the olfactory or trigeminal nerves that extend from the nasal cavity into the brain away from the BBB.

The DTE% was calculated for G-3 to assess the success of achieving brain targeting. As shown in Table 3, G-3 had DTE% of 141.42. Having DTE% > 100, superior drug delivery to the brain with the intranasal G-3 was achieved compared to the systemic administration. The favored brain drug delivery can be related firstly to the route of administration where the formula given by the intranasal route had the ability to be directly transported to the brain using the direct pathways of the olfactory and trigeminal nerves without having to cross the BBB while the intravenously administrated AGM did not have any direct pathway to the brain and it had to cross the BBB to reach it. The second reason for the betterbrain delivery was the form of AGM where the intranasal formula contained AGM loaded within SLNs while the intravenous formula contained AGM in the free form. As previously mentioned, the SLNs are expected to have better BBB uptake than the free drug.

Drug uptake into brain from the nasal mucosa occurred mainly via two different pathways. One was the systemic pathway by which some of AGM was absorbed into the systemic circulation and subsequently reached the brain by crossing the BBB. The other was the olfactory and the trigeminal neural pathway through which AGM directly traveled from the nasal cavity to CSF and/or brain tissue. To determine the relative contribution of the direct pathway in the overall brain delivery, the %DTP was calculated. Negative %DTP indicates more efficient drug delivery to the brain through the BBB permeation than the direct nose to brain routes while the positive values of the % DTE indicate a significant contribution of the direct routes to the overall brain delivery. Gel-3 gave DTP of 29.29 which revealed significant role of the direct pathway in the brain delivery for the two nasal formulae.

## Conclusion

Enhancement in both the absolute bioavailability and the brain delivery of AGM was achieved which can be attributed to the avoidance of the first pass metabolism due to the intranasal administration, the favored BBB uptake of the SLNs, the direct transport from nose to brain and the longer nasal rentention time due to the muciadhesive gel.
